# Pooled human bone marrow-derived mesenchymal stromal cells with defined trophic factors cargo promote dermal wound healing in diabetic rats by improved vascularization and dynamic recruitment of M2-like macrophages

**DOI:** 10.3389/fimmu.2022.976511

**Published:** 2022-08-19

**Authors:** Hélène Willer, Gabriele Spohn, Kimberly Morgenroth, Corinna Thielemann, Susanne Elvers-Hornung, Peter Bugert, Bruno Delorme, Melanie Giesen, Thomas Schmitz-Rixen, Erhard Seifried, Christiane Pfarrer, Richard Schäfer, Karen Bieback

**Affiliations:** ^1^ Institute of Transfusion Medicine and Immunology, Medical Faculty Mannheim, Heidelberg University, German Red Cross Blood Donor Service Baden-Württemberg - Hessen, Mannheim, Germany; ^2^ Institute for Anatomy, University of Veterinary Medicine Hannover, Hannover, Germany; ^3^ Institute for Transfusion Medicine and Immunohaematology, German Red Cross Blood Service Baden-Württemberg-Hessen, Frankfurt am Main, Germany; ^4^ Macopharma, Mouvaux, France; ^5^ German Society of Surgery, Berlin, Germany; ^6^ Institute for Transfusion Medicine and Gene Therapy, Medical Center – University of Freiburg, Freiburg, Germany; ^7^ Center for Chronic Immunodeficiency (CCI), Medical Center – University of Freiburg, Freiburg, Germany; ^8^ Mannheim Institute for Innate Immunoscience, Medical Faculty Mannheim, Heidelberg University, Mannheim, Germany; ^9^ FlowCore, Medical Faculty Mannheim, Heidelberg University, Mannheim, Germany

**Keywords:** mesenchymal stromal cells (MSCs), pooling, chronic wound healing, potency, efficacy, clinical scale production, platelet lysate, pathogen-inactivation

## Abstract

Human Mesenchymal Stromal Cells (hMSCs) are a promising source for cell-based therapies. Yet, transition to phase III and IV clinical trials is remarkably slow. To mitigate donor variabilities and to obtain robust and valid clinical data, we aimed first to develop a manufacturing concept balancing large-scale production of pooled hMSCs in a minimal expansion period, and second to test them for key manufacture and efficacy indicators in the clinically highly relevant indication wound healing. Our novel clinical-scale manufacturing concept is comprised of six single donor hMSCs master cell banks that are pooled to a working cell bank from which an extrapolated number of 70,000 clinical doses of 1x10^6^ hMSCs/cm^2^ wound size can be manufactured within only three passages. The pooled hMSC batches showed high stability of key manufacture indicators such as morphology, immune phenotype, proliferation, scratch wound healing, chemotactic migration and angiogenic support. Repeated topical hMSCs administration significantly accelerated the wound healing in a diabetic rat model by delivering a defined growth factor cargo (specifically BDNF, EGF, G-CSF, HGF, IL-1α, IL-6, LIF, osteopontin, VEGF-A, FGF-2, TGF-β, PGE-2 and IDO after priming) at the specific stages of wound repair, namely inflammation, proliferation and remodeling. Specifically, the hMSCs mediated epidermal and dermal maturation and collagen formation, improved vascularization, and promoted cell infiltration. Kinetic analyses revealed transient presence of hMSCs until day (d)4, and the dynamic recruitment of macrophages infiltrating from the wound edges (d3) and basis (d9), eventually progressing to the apical wound on d11. In the wounds, the hMSCs mediated M2-like macrophage polarization starting at d4, peaking at d9 and then decreasing to d11. Our study establishes a standardized, scalable and pooled hMSC therapeutic, delivering a defined cargo of trophic factors, which is efficacious in diabetic wound healing by improving vascularization and dynamic recruitment of M2-like macrophages. This decision-making study now enables the validation of pooled hMSCs as treatment for impaired wound healing in large randomized clinical trials.

## Introduction

Despite advances in patient stratification and treatments, chronic wounds are still an unmet clinical challenge for an increasing number of patients. Non-healing wounds are a particularly serious health problem for an aging population with severe comorbidities such as obesity, diabetes or cardiovascular diseases (1). The WHO reports 422 million patients with diabetes of whom 15-25% develop chronic wounds (https://www.who.int) (2).

Human Mesenchymal Stromal Cells (hMSCs) have been widely investigated in cellular therapies for the treatment of autoimmune, inflammatory, and vascular diseases (3, 4). Specifically, MSCs can improve wound healing, most likely by secreting factors associated with chemoattraction, cell proliferation and differentiation, immunomodulation, angiogenesis, anti-apoptosis, anti-fibrosis, and even anti-microbial effects (1, 5–7). This has led to several promising preclinical studies, as well as phase I and II clinical trials targeting chronic skin wounds, venous ulcers and epidermolysis bullosa (8–11). Yet, transition to phase III and IV clinical trials, or even marketing authorization, is remarkably slow. Next to safety and efficacy issues, often related to inconsistent study results, the so far tested hMSC therapies have been proven neither cost-effective, nor competitive against best-practice therapies (12). Next to technical obstacles (e.g. up-scaling and cryopreservation), issues pertaining to hMSC biology, such as donor variabilities, functional senescence, and the large variety of proposed mechanisms of action (MoAs), are increasing the complexity even further (13). Thus, to obtain robust and valid clinical data, it is of utmost importance to manufacture a substantial amount of hMSC doses from highly reproducible clinical hMSC products that can be tested in large randomized clinical trials. Furthermore, these products and their clinical evaluation require approval by the competent regulatory authorities. These expect a thorough scientific approach addressing GMP-compatible manufacturing, comprehensive quality control and in-depth preclinical efficacy and safety testing (13).

Upscaling issues of hMSCs were intensely discussed when a large phase III clinical trial failed to meet its clinical endpoint: the respective product “Prochymal™”, an allogeneic hMSC therapeutic, expanded *in vitro* to produce numerous clinical doses, lacked efficacy. In contrast, other allogeneic hMSC products, expanded to only few clinical doses, reproducibly showed efficacy in trials for steroid-refractory graft-versus-host-disease (GvHD) (14). This suggests that hMSCs manufacture should be carefully balanced to yield a sufficient number of clinical doses, but with only few population doublings during *ex vivo* production.

Inconsistent results from clinical trials may also result from donor-to-donor variability when hMSCs are manufactured from single donors (15). To address this, hMSC pooling concepts were developed. As one example, the product “MSC-FFM” was manufactured from pooled bone marrow (BM) mononuclear cells (MNCs), containing hMSC precursors as well as alloreactive immune cells of eight healthy 3rd-party donors (16). Besides reducing donor variability, an allogeneic immune reaction was intended to produce immunologically primed hMSCs with higher immunosuppressive strength. Indeed, for these cells beneficial effects in children and adults with severe steroid-refractory GvHD were reported (17, 18). Yet, highly immunosuppressive hMSCs may not be the first choice when aiming at chronic wound healing. Another example of a pooled hMSC product is “Stempeucel^®^”, successfully evaluated in critical limb ischemia. Here, a donor master cell bank (MCB) of single donor hMSCs after passage 1 was established (19). Next, a working cell bank (WCB) was generated by pooling hMSCs from three donors. This was then further expanded for five passages until the final product was cryopreserved. While the pooled “MSC-FFM” product requires establishing a new pooled hMSC MCB from scratch, the single-donor “Stempeucel^®^” hMSCs MCB concept allows high batch-to-batch consistency as recently shown (20). Yet, replicative aging within the five passages until reaching final product formulation may affect the quality of the clinical product (21).

To balance the needs for reproducible clinical results with consistent cell batches without extensive cell expansion, we developed a novel pooling concept based on various single donor MCBs, and pooled WCBs for final dose manufacture. We pooled six donors to efficiently level out donor-to-donor heterogeneity while keeping donor exposure low (22). In detail, we expanded the hMSCs from single donors in passage 0 and cryopreserved them as single donor MCBs. After thawing, we pooled single donor-derived hMSCs at different passages achieving a pooled hMSC MCB and expanded these as pooled hMSC WCBs up to passage 3. This concept allows identifying the pooled WCB with the best potential to manufacture maximal dose numbers at considerably low passage.

Human platelet lysate (hPL) is an increasingly used media supplement for cell therapies manufacture that promotes hMSC expansion *ex vivo* (23, 24). Yet, platelet donor variability and batch inconsistencies may hamper the implementation of robust manufacture concepts (13). To maximize consistency and ensure comparability, we used a large hPL batch pooled from 70 donors for the entire hMSC production series in our study. Pooling platelet donations equilibrates the hPL donor variability; yet, multi-donor exposure may increase the risk for transmitting infectious agents, which in turn can be addressed by pathogen reduction treatment (PRT) (22). Therefore, the pooled hPL batch used in this study was treated by high-dose gamma irradiation for pathogen reduction (25). The main advantages of gamma irradiation are that it does not involve any additives, and thus no residues, and that it can be directly applied to the final homogeneous and standardized pooled hPL batches. High-dose gamma irradiation (35 kGy) can efficiently inactivate both enveloped and non-enveloped viruses, meeting the current regulatory requirements (26), and irradiated hPL was shown to maintain hMSC proliferation capacity and function (25).

In addition to donor-to-donor variance and extensive expansion, cryodamage and dosing issues are discussed to compromise the success of hMSCs clinical translation. To ease manufacturing and delivery, hMSCs are typically expanded *ex vivo* and then cryopreserved as clinical products, which are then shipped to the patient´s bedside, where they are administered directly after thawing. Yet, cryopreservation may affect clinical potency associated with a heat-shock response, reduced immunomodulatory and homing capacity (27–29) and increased tissue factor expression (30).

To evaluate our novel hMSC product, we compared pools generated at passage 1, 2 and 3 (Pool 1, 2 and 3, respectively). Pools were first assessed *in vitro* for cell yield (clinical doses/batch), morphology, immune phenotype, growth factor content, proliferation, scratch/wound healing, chemotactic migration and angiogenic support. Based on highest yield and acceptable *in vitro* functions, we selected Pool 2 hMSCs for testing their wound healing capacity within a preclinical wound healing model *in vivo*. Zucker diabetic fatty rats were chosen as model of impaired and delayed wound healing (31). One million hMSCs/cm^2^ were topically applied within a diluted fibrin glue, previously shown to support cell viability and migration into the wounds (8, 9). In a series of pilot experiments, we compared possible cryodamage of freshly thawed hMSCs to hMSCs from rescue culture, and single versus 3-times repeated hMSC administration to target the different wound healing phases, respectively. Further, we assessed eventual systemic effects. The hMSC-treated wounds showed accelerated wound healing, accompanied by better wound indices (epidermal and dermal regeneration and collagen deposition), improved angiogenesis and increased macrophage infiltration and M2-like polarization.

## Methods and materials

### BM-derived hMSCs: Isolation, cultivation and characterization

**Figure 1:**
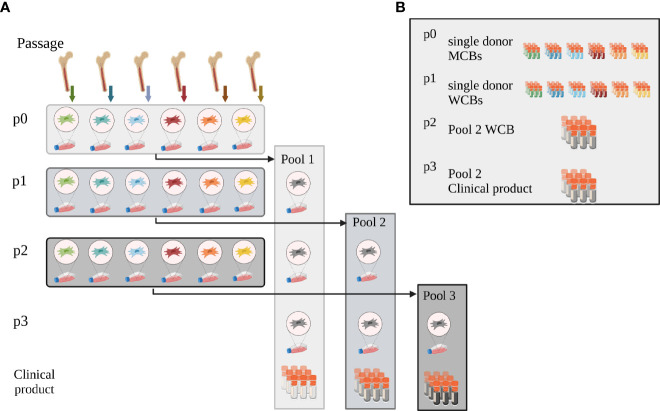
Pooling concept. **(A)** Pooling concept: hMSCs were isolated and expanded from bone marrow from six individual donors (passage p0). To initiate passage 1 expansion, single donor hMSCs were then thawed and either pooled at the onset of passage 1 (Pool 1) or expanded as single-donor hMSCs individually. These were then either pooled at the onset of passage 2 (Pool 2) or 3 (Pool 3), respectively. Pooled hMSCs at the end of passage 3 were cryopreserved, formulated as clinical product. **(B)** Master and Working Cell Bank concept: at the end of passage 0, single donor-derived hMSCs were cryopreserved as single donor MCBs. Single donor MSCs expanded in passage 1 were cryopreserved when harvested after passage 1 and served as WCBs. Pool 2 hMSCs, pooled at the onset of passage 2, were cryopreserved as working cell bank (WCB Pool 2) at the end of passage 2. Aliquots from this WCB were thawed and expanded one further passage to yield the potential clinical product end of passage 3. These cells were thawed and used for all experiments. Created with BioRender.com.

Human BM-MNCs were obtained by puncturing the iliac crest of healthy BM donors (ethical vote # 329/10, ethics committee, University Hospital Frankfurt am Main, Germany). The hBM-MNCs were seeded at a density of 100,000 cells/cm^2^ in Nunclon™ Delta flasks in 93% alpha-MEM with Glutamin (Lonza, Cologne, Germany), 6% pooled virally inactivated human platelet lysate (hPL) (MultiPL´100i, Macopharma, Tourcoing, France), 1% Penicillin/Streptomycin (Thermo Fisher Scientific, Darmstadt, Germany) and 2 IU Heparin (Ratiopharm GmbH, Ulm, Germany). After 24h, the non-adherent cells were removed by rinsing with PBS (Thermo Fisher Scientific) and culture medium exchange, and hMSCs were grown from the adherent cell fraction (15). For scale-up and GMP-compatible manufacture, hMSCs were also cultured in CellStacks with a larger culture surface enabling seeding, media exchange and harvest in a closed system (MC3 system, Macopharma). After reaching subconfluence, hMSCs were split using TrypLE™ Select (Thermo Fisher Scientific) and seeded at a density of 1,000 cells/cm^2^, or cryopreserved in 33% hPL, 5% DMSO (Sigma-Aldrich, Taufkirchen, Germany) in alpha-MEM as single donor-MCB. To level out individual differences, single donor hMSCs from six randomly chosen donors were then thawed and pooled at equal cell numbers (e.g. 6 times 1x10^6^ hMSCs), either at beginning of passage 1, 2 or 3 (Pool 1, 2 and 3, respectively). End of passage 3, hMSCs were cryopreserved as final (“clinical”) product. Pool 1 and 2 served as pooled WCBs (**Figure 1A**).

The hMSCs were verified to be mycoplasma- (Venor^®^ GeM Classic, Minerva Biolabs GmbH, Berlin, Germany) and endotoxin-free (Endosafe^®^ nexgen-PTS™, Charles River Laboratories, Freiburg, Germany).

Population doublings were calculated using the formula:


$$Population\;doublings\;(PD)=\frac{\text{ln(}\frac{harvest}{seed})}{ln2}$$

and maximum achievable cell number by


$$maximum\;achievable\;cell\;number=input*2^{population\;doubling}$$

Maximum achievable cell numbers at end of passage 3 and target cell dose equivalents (1x10^6^ hMSCs/cm^2^ wound size) were extrapolated for Pools 1 -3, respectively (32).

The hMSCs were characterized by a battery of *in vitro* test systems: First, marker expression (binary markers, either absent or present on hMSCs (33)) was assessed by flow cytometry (32). Second, adipogenic differentiation was induced using the hMSC Adipogenic Differentiation Medium BulletKit™ (Lonza, Basel, Switzerland) and osteogenic differentiation using osteogenic medium composed of alpha-MEM, 10% FBS supplemented with 1 µM dexamethasone, 50 µM ascorbic acid and 10 mM β-glycerolphosphate (Sigma-Aldrich), respectively. After three weeks of differentiation cells were lineage specifically stained: lipid vacuoles in adipogenic differentiation cultures were stained with Oil Red O, calcium deposits of osteogenic differentiated cells with Alizarin Red, respectively. Third, the hMSCs’ capacity to inhibit T cell proliferation *in vitro* was assessed (32). Briefly, hMSCs were pre-seeded and CellTrace™ Violet (Thermo Fisher)-labeled pooled peripheral blood mononuclear cells (PBMNCs) were added, and further stimulated with phytohemagglutinin-L (PHA-L, 10 µg/mL, Sigma-Aldrich), or kept as non-stimulated controls. Proliferation of PBMNCs was assessed after 5 days using flow cytometry and hMSC-mediated inhibition was calculated. Fourth, live cell imaging using the Incucyte^®^ Zoom device (Sartorius AG, Hertfordshire, United Kingdom) was performed and analyzed using Incucyte^®^ analysis algorithms. First, hMSCs proliferation was assessed by seeding 200 hMSCs/cm^2^ and monitoring the increase in cell confluence over time. The 96h time point was chosen to compare Pools 1-3. Second, a scratch wound healing assay of hMSC monolayer was performed. In detail, hMSCs were seeded at 60,000 cells/cm^2^ and incubated overnight. Then, wound scratches were applied using the 96-pin Incucyte^®^ woundmaker tool. The wound closure (wound density at different time points relative to initial wound size) was calculated over time and values at 24h used for comparison. Third, angiogenic tube network formation on hMSCs monolayers was assessed, as described previously (34). hMSCs were seeded at 60,000 cells/cm^2^ and after 60min 15,000 green fluorescent protein (GFP)+ human umbilical vein endothelial cells (HUVECs) were added. Human adipose-derived stromal cells (hASCs) served as positive control and were used for normalization of individual experiments. Network length (mm/mm^2^) was chosen as parameter for quantitative analysis. Fourth, chemotactic migration of hMSCs was assessed (35). Briefly, the insert plate of an Incucyte^®^ ClearView 96 well plate was coated with fibronectin. Subsequently, 1,000 hMSCs were seeded and the plate was mated with the reservoir plate containing serum-free or hPL-containing medium. hMSCs migration was monitored for 48h and analyzed as “count normalized to initial top value”.

Trophic factors of hMSCs lysate were quantified using Luminex and ELISA technologies (32). Briefly, 1-10 x 10^6^ hMSCs were harvested. hMSC pellets were lysed with ice-cold ProcartaPlex™ Cell Lysis Buffer, centrifuged at maximum speed and supernatant stored at -80°C until assays were performed. Transforming growth factor beta 1 (TGF-β1) and Prostaglandin E2 (PGE2) were analyzed by ELISA (Biorbyt Ltd., Cambridge, UK and Cayman Chemical, Ann Arbor, MI, USA, resp.), Fibroblast growth factor 2 (FGF2) was analyzed by singleplex and all other trophic factors using a ProcartaPlex™ custom multiplex panel (Thermo Fisher Scientific).

hMSCs’ IDO-1 production was stimulated by tumor necrosis factor- α (TNF-α), interleukin-1β (IL-1β), and interferon-ɣ (IFN-ɣ), each 20 ng/mL for 48h. Subsequently, hMSCs were harvested and counted. Pellets were lysed (300 mM NaCl, 50 mM Tris, 2 mM MgCl_2_, 0.05% NP40, 1x Protease/Phosphatase Inhibitor), centrifuged at maximum speed and supernatant stored at -80°C until ELISA (Cloud-Clone Corp., Katy, TX, United States) was performed.

To assess the trophic factors content in the pooled hPL batch used in this study (MultiPL’100i; batch number 11219267DM), two different bags were tested by ELISA (Bio-techne; FGF (#SFB50), vascular endothelial growth factor-A (VEGF-A; #SVE00), epidermal growth factor (EGF; #SEG00), platelet-derived growth factor-AB (PDGF-AB) (#SHD00C), insulin-like growth factor-1 (IGF-1) (#SG100) and TGF-β1 (#SB100B)).

### Wound healing model

**Figure 2:**
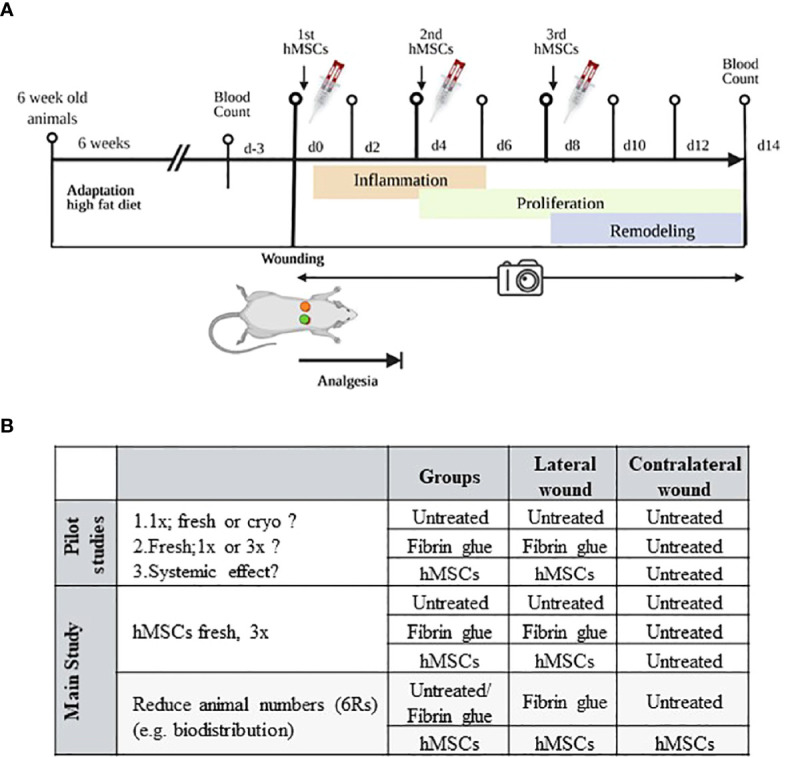
Schematic of *in vivo* wound healing assay. **(A)** Diabetic ZDF rats were wounded and treated topically with hMSCs (1x10^6^/cm^2^ wound) in diluted fibrin glue. Untreated and cell-free fibrin glue-treated wounds served as control (created with BioRender.com). **(B)** Table representing animal allocation. fresh, MSCs from max. 2 days of rescue-culture before administration to the wounds; cryo, MSCs thawed immediately before application; 1x, single application day 0; 3x, repeated application d0, day 4 and day 8.

Animal experiments were approved by the local ethics committee (G142-19, Regierungspräsidium Karlsruhe, Germany). Zucker diabetic rats were chosen as model of impaired wound healing (31). In total, 66 six weeks old male rats (ZDF (obese fa/fa), ZDF-Leprfa/Crl; Charles River Laboratories, Châtillon, France) were used. Upon arrival, rats were kept in groups of two and fed ad libitum with a special high-fat diet (Purina #5008, ssniff Spezialdiät GmbH, Soest, Germany) for 6 weeks to induce diabetes type II (**Figure 2A**). Rats were weighed every 2 days and non-fasted blood glucose was measured once a week (Accu-Chek^®^ Aviva, Roche Diabetes Care, Mannheim, Germany). Animals were considered diabetic with a glucose level of 300 mg/dL, typically reached 3 weeks after initiating diet. Only diabetic rats were used for the wound healing experiments. Rats with blood glucose levels above 600 mg/dL were fed with the normal food until blood glucose levels dropped.

At 12 weeks of age, rats were anaesthetized with isoflurane (CP-Pharma 1 mL/mL, induction with 5% isoflurane plus 5 L/min oxygen and maintenance with 2-3% isoflurane plus 1 L/min oxygen) and 0.8 mL of blood were taken. For wound setting the rats were shaved on the back, the surgery field was disinfected and two wounds were set 1.5 cm behind the shoulder blades and 1.5 cm right and left from the spine with an 8 mm skin biopsy punch (WDT^®^, Garbsen, Germany). Only the skin was removed, the skeletal muscle fascia was left intact. Depending on the experimental setting, control animals were either left completely untreated or one wound was untreated and the other treated with cell-free fibrin glue. In other animals, one wound was treated with fibrin glue plus hMSCs, whereas the contralateral wound served as control, left untreated or treated with cell-free fibrin glue (**Figure 2B** **)**. Post-surgery 10 mL of physiological saline was injected subcutaneously to avoid dehydration and allow faster recovery after anaesthesia. To protect wounds from contamination or mutilation, a wound dressing was applied (Curapor^®^, Lohmann-Rauscher, Rengsdorf, Germany). This dressing was changed every other day. 200 mg/kg of metamizole sodium was used for analgesia (Novaminsulfon^®^ solution for injection 500 mg/mL, Bela-pharm, Vechta, Germany) given for 4 days by subcutaneous injection.

For topical cell application, a commercial fibrin sealant syringe system was used (TISSEEL, Baxter Deutschland GmbH, Unterschleißheim, Germany). hMSCs were either thawed (cryo) or trypsinized after a short rescue culture (fresh; cells were thawed, cultured for up to two days to recover from eventual cryo-damage and to re-boot their metabolism), washed, counted and formulated at a density of 5x10^5^ viable hMSCs in 50 µL prediluted fibrinogen/aprotinin solution (final concentration 5 mg/mL) (9). Immediately before the application to the wounds, the cell suspension was drawn in one syringe of the duplojet device, while the other syringe contained prediluted thrombin solution (final concentration 25 mg/mL). Both components were combined using the TISSEEL duplojet system to formulate the fibrin glue. For each wound, 50 µL fibrinogen with hMSCs and 50 µL thrombin were then applied onto the wound, resulting in a dose of 1x10^6^ hMSCs/cm^2^ wound. The glue was allowed to polymerize in the air for 7 minutes before applying the wound dressing.

To assess the capacity of the hMSCs to migrate out of the gel, an *in vitro* migration assay was performed. Briefly, fibrin glue with hMSCs was applied into a well of a 24-well plate. Subsequently, the hMSCs` migration towards hPL-supplemented culture medium as attractant, or serum-free medium as control, was evaluated microscopically.

Three pilot studies were performed, according to the 6R principles with each 3-5 animals. First, to pretest eventual cryopreservation damage we compared freshly thawed (cryo) hMSCs against rescue culture post-thaw (fresh) hMSCs (28–30). Second, we compared single dose injection (d0) against repeated hMSCs administration (days 0, 4 and 8) to apply hMSCs at the inflammation, proliferation and remodeling phase respectively (**Figure 2A**). Of note, despite smaller wound sizes, we applied the same cell dose as on d0. Third, we investigated eventual systemic effects of hMSCs. Here, single rats were allocated into one group where the contralateral site served as control, and the lateral site was treated with hMSCs and compared to a group of animals with only control-treated wounds.

With the results from the pilot study, a power analysis was performed to calculate the sample size for the main study. Here, culture-adapted fresh hMSCs were applied repeatedly, but treatments of the two wounds were chosen randomly (in total, n= 42 rats in the main study). To overcome potential breed-specific biases, experiments were performed in different experimental cohorts.

Furthermore, a biodistribution study was performed where animals were sacrificed on day 1, 2, 3, 4, 9 and 11 (each one animal per group). Here, both wounds served as either control (untreated/fibrin group) or were hMSCs-treated. Wound, liver, spleen and lungs were harvested, snap-frozen and then analyzed for the presence of human cells by immunohistochemical staining and digital PCR (dPCR).

For each animal, every other day upon wound dressing change, the wounds were scaled and photographed with a perpendicular angle. Wound area was measured using ImageJ (36). In addition, blood samples were taken after 14 days before the animals were sacrificed and a blood count performed (CELL-DYN Ruby, Abbott GmbH, Wiesbaden, Germany). First, the rats were fully anaesthetized with isoflurane and then 100 mg/kg ketamine and 5 mg/kg xylazine were injected intracardially. To avoid autolysis, wounds and organs were removed immediately. Wounds were cut in half to perform all analyses at the wounds center and were either paraformaldehyde (PFA)-fixed and paraffin-embedded or snap-frozen in Tissue-Tek^®^ and cryomolds.

#### Histological and immunohistochemical analysis

Standard hematoxylin-eosin (HE) and Azan staining was performed on 5 µm thick cuts after organ fixation in 4% PFA and paraffin embedding.

To investigate whether hMSCs promote host cell infiltration into the wound, artificial intelligence and QuPath algorithms (37) based on a random tree classifier were used for analyzing HE stains. First, the wound was defined as region of interest. Second, an automatic cell detection was run to determine the total cell count in this area. Using QuPath-based cell classification, fibroblasts and lymphocytes were discriminated based on nuclear stain (more homogenous and intense in lymphocytes than fibroblasts) and cellular morphology (round lymphocytes versus elongated fibroblasts). In addition, a “composite classifier” was used to improve the differentiation of lymphocytes characterized by their very pronounced circularity compared to fibroblasts.

Heidenhain’s Azan trichrome stain was performed to assess collagen fiber deposition. Mean blue intensity was taken as measure of collagen density and dermis maturation. For this the “intensity mean value: blue” feature was used (Zeiss Zen 3.0 blue edition, Carl Zeiss Microscopy, Oberkochen, Germany). This tool calculates the average brightness (pixel value) of the selected region of interest. Dark blue colors reflecting high collagen density have lower pixel values than the lighter blue stains of wounds with fewer collagen fibers. Pixel values of the wound tissue were compared with those of the surrounding not injured dermis, with lower intensity equivalent to more collagen deposition in the granulation tissue.


$$\text{Blue mean intensity in \%}=\frac{wound intensity mean blue value * 100}{\text{not injured dermis intensity mean blue value}}$$

Immunohistochemical staining was performed to assess the degree of vascularization (CD31+ endothelial cells), immune cell infiltration (CD68+ and CD163+ macrophages) and presence of the transplanted hMSCs (human Ku80+ cells (38)). Cryosections (10 µm) were fixed in 4 % PFA for 10 minutes. Nonspecific binding sites were blocked using 1 % bovine serum albumin (BSA, PAN-Biotech, Aidenbach, Germany), 0.2 % fish skin gelatin (Sigma-Aldrich) and 0.1 % Triton X (Carl Roth, Karlsruhe, Germany) in Tris-Buffer saline. Antibodies were then added and incubated overnight (each 1:1,000 for mouse anti-rat monoclonal CD31 (Ab64543), Abcam, Cambridge, UK, rabbit anti-rat polyclonal CD68 (Ab125212) Abcam, mouse anti-rat monoclonal CD163 (MCA342GA) BioRad, Feldkirchen, Germany, and 1:250 rabbit anti-human monoclonal Ku80 (EPR3468), Abcam). After washing, endogenous peroxidase was blocked in 3 % H_2_O_2_. Then, the secondary biotinylated antibody was added for 30 min (1:100 anti-mouse and anti-rabbit Ig, (RPN1001V, RPN1004V1) GE-healthcare, Solingen, Germany). Then 1 % streptavidin peroxidase (GE-healthcare) was added. Histogreen was used as substrate chromogen (Linaris GmbH, Dossenheim, Germany). Nuclei were counterstained with Mayer’s hematoxylin and sections mounted after dehydration in 99 % ethanol, tissue clear and n-butyl acetate. Control slides were either left unstained to evaluate Histogreen background signal or stained with only the 2^nd^ antibody. Slides were scanned (Zeiss AXIO Scan.Z1) and analyzed using QuPath open software (36), creating a color filter to quantify histogreen-positive area in the entire wound previously defined as region of interest.

hKu80 staining in the organs was validated using Alexa Fluor 488- or Alexa Fluor 568-labeled secondary antibodies, (1:1,000; Life Technologies, Thermo Fisher Scientific) and TO-PRO-3 nuclear stain (Thermo Fisher Scientific) and assessed by confocal microscopy.

#### Histology scoring system

##### Epidermal Thickness Index (ETI)

In 14 days old wounds, the average thickness of the wound epidermis was calculated for five locations and compared to the average thickness of the non-lesioned epidermis.


$$\;ETI=\frac{average epidermis thickness in wound area * 100}{\text{average epidermis thickness in uninjured skin}}$$

An ETI > 105 % is considered hypertrophic and mostly observed during the re-epithelialization phase and is an indicative of healing. A return of the epidermis thickness close to non-injured skin (95%<ETI<105 %) is only observed after remodeling stage (39).

##### Scar elevation Index (SEI)

In 14 days old wounds, the average thickness of the dermis was calculated using five areas and compared to the average thickness of the unwounded dermis.


$$SEI=\;\frac{average dermis thickness in wound area * 100}{\text{average dermis thickness in uninjured skin}}$$

A hypertrophic dermis in the wound (SEI>105 %) can reflect excessive collagen deposition and is therefore an indirect indicator of scar formation. A hypotrophic dermis with a SEI<95 % is typically reported in early stages of healing wounds and reflects an underdeveloped dermis. A 95<SEI<105 % characterizes a wound dermis whose thickness has returned to normal and is only observed in the final stage of healing (39).

#### Chip-based dPCR to detect residual human cells

To follow the fate of the topically applied hMSCs, wounds and organs were analyzed for human DNA using a sensitive dPCR method (40).

The dPCR assay was designed for detection of the single locus gene *GAPDH* in the rat and the human genome with specific primers and TaqMan™ probes with minor groove binding (MGB) modification at the 3’-end. For human *GAPDH*: forward primer, 5’-ccccacacacatgcacttacc-3’; reverse primer, 5’-cctagtcccagggctttgatt-3’; VIC-labeled probe, 5’-taggaaggacaggcaac-3’; for mouse/rat *GAPDH*: forward primer, 5’-gaatataaaattagatctctttggac-3’; reverse primer, 5’-gttgaatgcttggatgtacaacc-3’; FAM-labeled probe, 5’-taggaaggacaggcaac-3’. The human/rat GAPDH assay was prepared as 40x concentrated mixture containing 9 µmol of each primer and 5 µmol of each probe resulting in a final concentration of 225 nmol of each primer and 125 nmol of each probe.

The dPCR (QuantStudio^®^ 3D; Thermo Fisher Scientific) was performed on chips with 20,000 reaction wells each with 755 pL volume. For each dPCR analysis 7.1 µL DNA was mixed with 0.375 µL 40x GAPDH assay and 7.5 µL dPCR Master Mix V2 containing ROX as reference dye (Thermo Fisher Scientific). The cycling program started with 10 min at 96 °C, followed by 40 cycles with 30 sec at 98 °C and 2 min at 52 °C. After cycling the dPCR chips were scanned for the FAM and VIC signals (QuantStudio^®^ 3D Chip Reader; Thermo Fisher Scientific) and the data were analyzed using the QuantStudio 3D AnalysisSuite cloud software (https://apps.thermofisher.com/quantstudio3d). Based on the fluorescence signals and statistical correction using Poisson distribution the software enabled calculation of target copies per µL and target/total (%) values. For validation of the assay, human and rat genomic DNA was used pure and mixed at defined ratios (1:10, 1:20, 1:50). Human DNA was reliably detectable in the 1:50 mixture (detection limit 2 %; approx. 4 copies/µL), whereas, pure rat DNA showed a background signal of 0.2% (approx. 0.4 copies/µL). 0.5 copies/µl were calculated as cut-off for positive signals.

### Statistics

Quantitative data are presented as means ± standard deviation (SD) and were compared with analysis of variance (ANOVA) and *post hoc* tests as specified in the figures using GraphPad Prism (La Jolla, CA, USA). Values of *p*< 0.05 were considered as statistically significant.

## Results

### Closed system and pooled hPL allow scale-up manufacture of pooled hMSC doses with defined trophic factors content

For scale-up and GMP-compatible manufacture, hMSCs were simultaneously cultured in standard Nunclon™ Delta flasks (175 cm² per flask) as well as in CELLSTACK™ with a larger culture surface (636 cm² per stack) enabling seeding, media exchange and harvest in the closed MC3 system. Growth kinetics and hMSC surface marker expression were identical (not shown). Production of clinical-scale doses was more feasible with the closed MC3 system due to optimized handling for media changes and passaging/harvest particularly reducing hands-on time in the cell culture.

**Figure 3:**
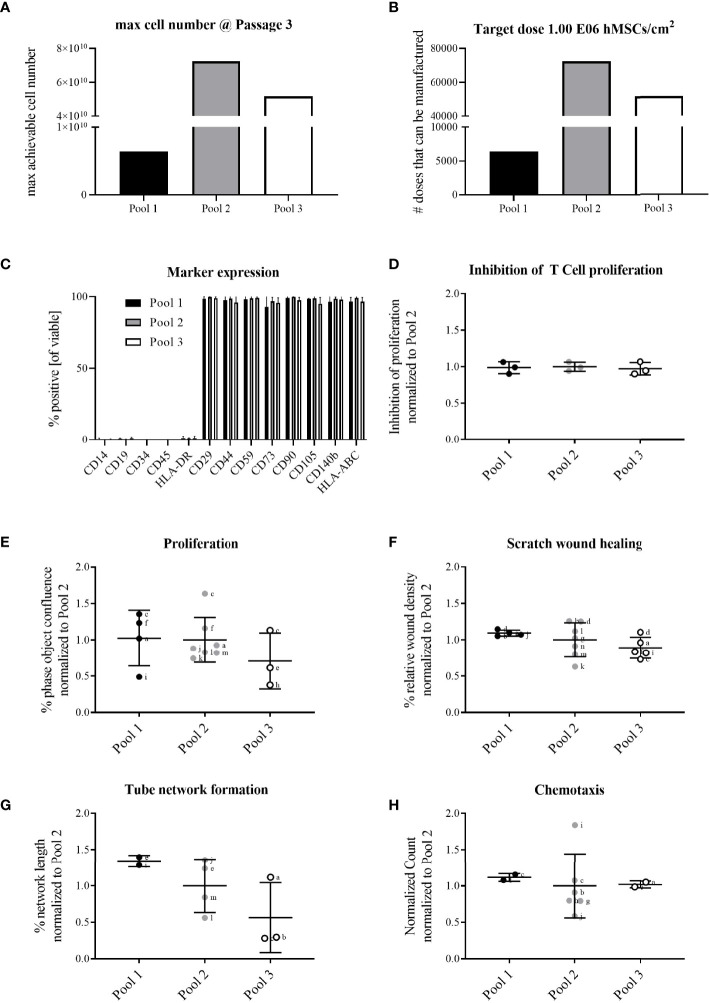
All hMSC pools featured similar proliferation capacity, yet with Pool 2 hMSCs the highest number of extrapolated clinical doses could be achieved. All pools exerted similar functional characteristics. Calculation of **(A)** maximally achievable cell doses/manufacturing batch and **(B)** cell doses manufactured/batch (1x10^6^ hMSCs/cm^2^ wound size) for hMSCs pooled at either passage 1 (Pool 1), passage 2 (Pool 2) or passage 3 (Pool 3). **(C)** Flow cytometry characterization of binary (absent or present) hMSCs markers. **(D-H)** Functional characterization of hMSC pools. Data are shown as normalized to the average of Pool 2. **(D)** hMSCs-mediated inhibition of PHA-driven T cell proliferation. **(E–H)** Live cell imaging analyses of functional hMSC attributes: **(E)** proliferation; phase object confluence 96h post-seeding; **(F)** scratch wound healing: relative wound density at 24h post-wounding; **(G)** tube network formation: hMSCs were seeded as monolayer and fluorescently-labeled endothelial cells (HUVEC) were seeded on top. Tube length was assessed 48h post-seeding and calculated as percent of human adipose stromal cell-mediated tube formation; **(H)** chemotactic migration to hPL-supplemented medium in bottom chamber. Counts of migrated cells in bottom wells were normalized to initial top-well values. Serum-free medium served as negative control. **(E–H)** Small letters indicate experimental replicates performed on different days by different operators. All data are shown as data from individual experimental replicates, indicating mean ± SD.

The extrapolated maximum of cell numbers that could be produced in line with highest number of target cell doses (~70,000 extrapolated doses) was achieved with Pool 2 hMSCs compared to Pool 1 (~6,000 extrapolated doses) and 3 (~50,000 extrapolated doses) hMSCs (**Figures 1A, B**, **3A, B**). For all hMSC pools, the expression of binary (absent or present) MSC markers was identical, consistent with guidelines set by the International Society for Cellular Therapy (**Figure 3C**). The functional characterization of the hMSC pools proved similar regarding their adipogenic and osteogenic differentiation potential **(** **Supplementary Figure 1** **)** and their immunomodulatory strength measured by their inhibition of PHA-driven T cell proliferation (**Figure 3D**). Proliferation, scratch wound healing, vascular tube formation support and chemotactic migration assessed by live cell imaging showed also no differences between the hMSC pools (**Figures 3E–H**). Yet, due to apparent day-to-day and operator-to-operator related variations in the latter assays, the need for better assay standardization became obvious.

Given that Pool 2 hMSCs achieved the highest calculated numbers of extrapolated clinical doses with similar characteristics compared to Pool 1 and 3 hMSCs, we elected Pool 2 hMSCs for further preclinical evaluation.

The delivery of trophic factors is a key MoA of hMSCs (13). Therefore, we quantitatively evaluated trophic factor candidates for wound healing. Of note, we analyzed the hMSC lysates reflecting the actual clinical product, rather than mere cell culture supernatant collected during expansion. Specifically, we detected BDNF, EGF, G-CSF, HGF, IL-1α, IL-6, LIF, osteopontin, VEGF-A, FGF-2, TGF-β, PGE-2 and inducible IDO-1 in the hMSCs and calculated their contents per applied hMSC dose (**Table 1**). GM-CSF, IL-1β, NGF-β, angiopoietin, IFN-ɣ, IL-2 and TNF-α were below the detection limit of the assay. These growth factors are active in different phases of wound healing.

**Table 1:** Trophic factors in Pool 2 hMSC lysate, calculated per hMSCs dose.

			
	pg/applied hMSCs dose	Wound healing function	References
**Luminex**
BDNF	3.06	Acts proangiogenic	(41)
EGF	0.58	Induces migration, proliferation, plasticity of epithelial cells, fibroblast function, formation of granulation tissue	(42, 43)
G-CSF	1.99	Accelerates wound healing, promotes neutrophil infiltration	(44)
HGF	122.68	Induces migration, proliferation, and matrix metalloproteinase production of keratinocytes, acts proangiogenic	(42, 45)
IL-1*a*	2.33	Stimulates keratinocyte and fibroblast proliferation, extracellular matrix remodeling, fibroblast chemotaxis, regulates the immune response	(46)
IL-6	23.08	Mitogenic for keratinocytes, promotes neutrophil attraction	(42)
LIF	6.94	Enhances proangiogenic potential of hMSCs	(47)
Osteopontin	21.63	Regulates ECM, myofibroblast differentiation	(41, 42)
VEGF-A	174.43	Acts proangiogenic	(41)
FGF2	2.69	Acts proangiogenic, mitogenic for fibroblasts and keratinocytes	(42)
GM-CSF	below detection limit	Mitogenic for keratinocytes, induces migration and proliferation of endothelial cells, regulates macrophage polarization	(41, 42)
IL-1β	below detection limit	Acts proinflammatory	(41)
NGF-*b*	below detection limit	Stimulates nerve ingrowth	(42)
Angiopoietin	below detection limit	Induces vessel stabilization and remodeling	(42)
IFN-γ	below detection limit	Modulates cell-mediated immunity, neutrophil inflammatory response, M1 polarization, can impair wound healing	(48, 49)
IL-2	below detection limit	Attracts immune cells	(50, 51)
TNF-α	below detection limit	Proinflammatory, inhibits myofibroblast differentiation	(41)
**ELISA**			
			
TGF-*β*1	162.29	Promotes chemoattraction, angiogenesis, M2 macrophage polarization, myofibroblast differentiation, mitogenic for fibroblasts, inhibits proliferation of keratinocytes, stimulates ECM proteins and integrin expression	(41, 42)
PGE2	26.57	Induces anti-inflammatory responses, M2 macrophage polarization, is proangiogenic, reduces pathological scar formation	(52)
IDO-1 (after stimulation for 48h with TNF-*a*, IL-1β, IFN-γ)	555.07	Modulates immune responses	(53)

BDNF, brain-derived neurotrophic factor; EGF, epidermal growth factor; G-CSF, granulocyte colony stimulating factor; HGF, hepatocyte growth factor; IL-1*a*, interleukin 1 alpha; IL-6, interleukin 6; LIF, leukemia inhibitory factor; VEGF-A, vascular endothelial growth factor A; FGF2, fibroblast growth factor 2; GM-CSF, granulocyte-macrophage colony-stimulating factor; IL-1β, interleukin 1 beta; NGF-β, nerve growth factor beta; IFN-γ, interferon gamma; IL-2, interleukin 2; TNF-*a*, tumor necrosis factor alpha; TGF-β1, transforming growth factor beta 1; PGE2, prostaglandin E2; IDO-1, indoleamine 2,3-dioxygenase.

Given that the media supplement influences the final trophic factors composition of the hMSC lysates (54), we tested also the hPL batch used in this study (**Supplementary Table 1**). Here, we detected high concentrations of TGF-β1, EGF, PDGF-AB and VEGF-A, mirrored by the relatively high amounts of TGF- β1 and VEGF-A in the hMSC dose (**Table 1**).

### hMSCs migrate from the fibrin glue and improve skin wound healing in diabetic rats

For cell application, we used a protocol established by Yufit et al. using 1:10 diluted TISSEEL fibrin glue as cell carrier (9). In a pilot *in vitro* experiment, we verified that hMSCs formulated in the 1:10 diluted fibrin glue were able to egress and migrate from the glue. The diluted fibrin glue needed about 7 minutes to polymerize to a gel. After 4.5 hours the hMSCs started to migrate from the glue into the culture vessel, and hMSC migration increased over time (**Supplementary Figure 2**). Of note, no migration was induced in serum-free conditions indicating targeted migration of hMSCs.

For the *in vivo* evaluation of the wound healing potential of pooled hMSCs, three pilot studies were performed in preparation for the main study. In each study, two circular wounds of 8 mm diameter were set per one animal and either left untreated, treated with cell-free fibrin glue, or with hMSCs-formulated fibrin glue (**Figures 2A, B**).

**Figure 4:**
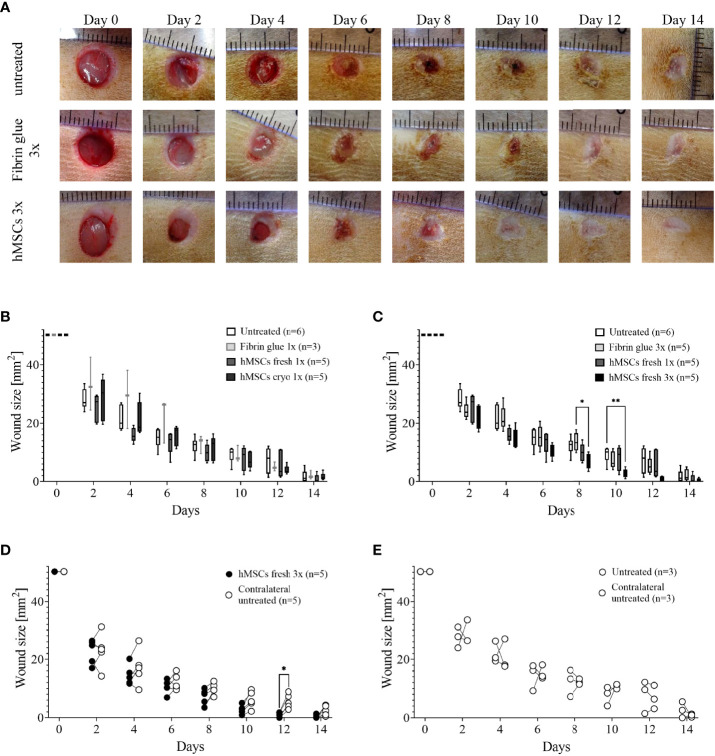
Pilot studies 1-3 to assess cryodamage, dose finding and systemic wound healing effects. **(A)** Representative images of wounded skin after single topical treatment with either hMSCs in fibrin glue, cell-free fibrin glue or untreated at d0 after wounding. **(B)** Wound area reduction after topical treatment with either untreated, cell-free fibrin glue and hMSCs thawed (cryo) or from rescue culture (fresh), **(C)** Single versus 3-times repeated hMSCs administration (1x versus 3x fresh, d0, 4 and 8). Quantification of wound areas relative to initial wound area was performed with Image J. Data are presented as min to max box-whisker plots denoting the median. **(D, E)** Comparison of wound size reduction of contralateral untreated wounds with lateral either hMSCs **(D)** or untreated wounds **(E)**. Side-by side comparison of hMSCs fresh vs. contralateral untreated and untreated vs. contralateral untreated shown. *p≤ 0.05, **p≤ 0.01 as calculated using two-way ANOVA und Tukey multiple comparisons. fresh, MSCs from max. 2 days of rescue-culture before administration to the wounds; cryo, MSCs thawed immediately before application; 1x, single application day 0; 3x, repeated application d0, day 4 and day 8.

In pilot study 1, we evaluated an eventual cryodamage comparing just thawed hMSCs (cryo) with hMSCs from a rescue culture (fresh). From day 4 on, wounds treated with fresh hMSCs healed slightly better than hMSCs cryo (**Figure 4B**). On day 12, a significantly smaller wound size was calculated in hMSC cryo-treated wounds compared to untreated wounds. Cell-free fibrin glue per se, compared to untreated wounds, promoted wound healing, but slightly delayed compared to hMSCs-treated wounds (d10 and d12, **Figure 4B**). Based on these data, we concluded that hMSCs show a slight cryodamage and favored the use of rescue-cultured fresh hMSCs for the subsequent experiments.

In pilot study 2, we evaluated whether repeated application on days 0, 4 and 8 of fresh hMSC doses could further accelerate wound healing. Given that the hMSC therapeutic contained a large variety of growth factors, known to be active, and thus, being required, during the inflammation, proliferation and remodeling phase of wound healing, we applied hMSCs at respective time points, d0, d4 and d8 reflecting the different wound healing phases (**Figure 2A**). Starting at day 4, wounds treated with hMSCs trended smaller than control-treated wounds, but from day 8 on, wounds treated 3 times with hMSCs were significantly smaller compared to controls (**Figures 4A, C**).

### Topically applied hMSCs do not exert systemic wound healing effects

Further, blood samples collected during the pilot studies were analyzed comparing white blood cells (WBCs), neutrophil, lymphocyte and platelet counts on day 0 and day 14. In the control settings, all blood cell counts appeared to be increased at day 14. Yet, WBCs and especially lymphocyte counts in 9 out of 15 hMSCs-treated animals were decreased compared to d0. This effect was more pronounced after repeated hMSCs application (not shown). None of the control animals showed this trend.

Accordingly, we asked in pilot study 3 whether hMSCs would exert systemic effects and could affect healing of the contralateral wound where no hMSCs were applied topically. Statistical analysis revealed that only the wounds that were topically treated with three hMSC doses at the different time points significantly improved healing. The contralateral untreated site showed comparable healing as untreated wounds (**Figures 4D, E**; **Supplementary Figure 3**). These data suggest that topically applied hMSCs exert their therapeutic wound healing effects only locally.

Based on the results from these pilot studies and a power-based sample size calculation, the main study was designed (i) using fresh, rescue-cultured hMSCs, (ii) repeated application of hMSC doses on day 0, 4 and 8, and (iii) reduced numbers of control wounds according to the 6Rs principles, as systemic effects were excluded.

**Figure 5:**
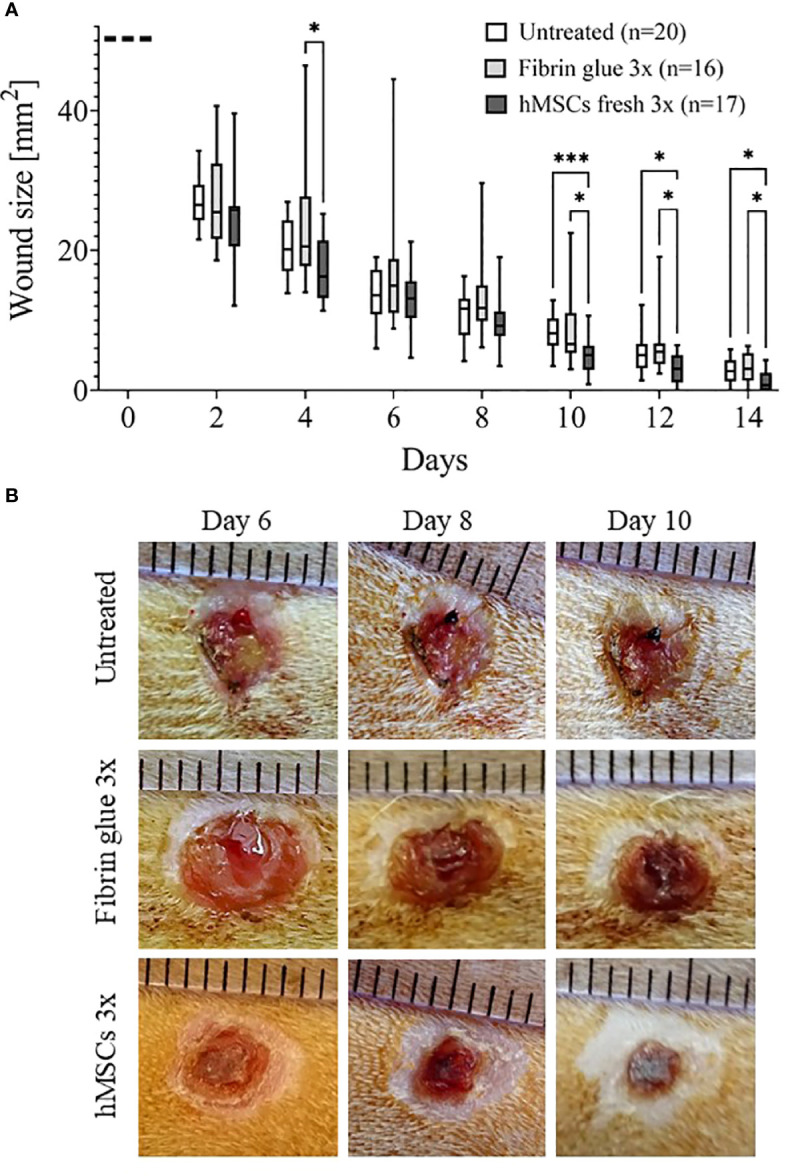
hMSCs improve wound healing. **(A)** Wound size reduction after 3-times repeated topical treatment with either hMSCs (1x10^6^/cm^2^) in fibrin glue, cell-free fibrin glue or untreated at d0, d4 and d8 after wounding. Quantification of wound areas relative to initial wound area was performed with Image J. * p≤ 0.05, *** p≤ 0.001, as calculated using two-way ANOVA and Tukey multiple comparisons. **(B)** Representative images of wounded skin after 3-times repeated topical treatment with either hMSCs in fibrin glue, cell-free fibrin glue or untreated depicting crust formation, especially in fibrin glue-treated wounds.

Results from the main study supported the significant wound healing effect of hMSCs. Wounds treated with three sequential hMSC doses were significantly smaller on days 10, 12 and 14 compared to both controls (**Figures 5A, B**). Importantly, some wounds treated with hMSCs were already closed on day 12 after wound setting. In both control groups the first wounds were completely healed only at day 14. We found that both control wounds, untreated and cell-free fibrin glue-treated, showed similar wound healing rates. In these series of experiments, we observed extensive crust formation in fibrin glue-treated wounds, but not the hMSC-fibrin glue-treated wounds (**Figure 5** **)**.

The data from the pilot and the main studies documented that hMSCs significantly improved wound healing compared to both control groups. Yet, the initially observed trend of decreased circulating lymphocytes within peripheral blood after topical hMSCs application was not confirmed.

### hMSCs increase CD31-positive capillaries and recruit CD68- and CD163-positive macrophages into healing wounds

**Figure 6:**
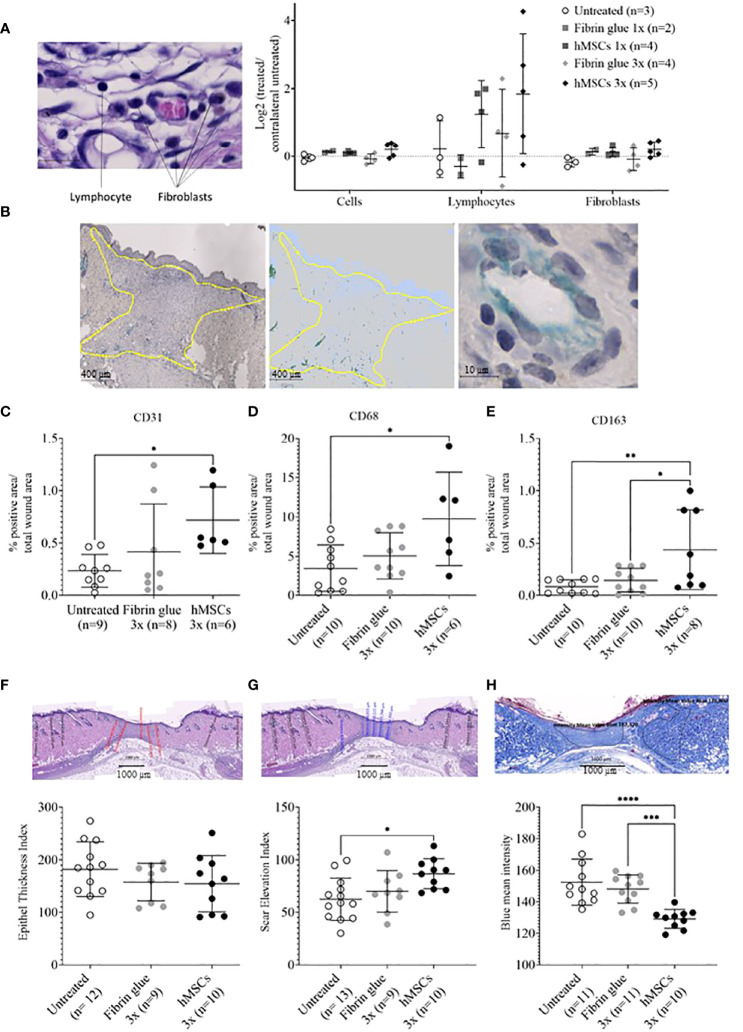
hMSCs tend to increase lymphocyte infiltration, CD31+ vascularization and CD68- and CD163-positive macrophage infiltration, and to improve wound healing indices. Wound skin was harvested at d14 and histologically analyzed. **(A)** Frequencies of total cells, lymphocytes and fibroblasts within wounds analyzed by QuPath algorithm on HE stains. Values were normalized against the untreated contralateral site. **(B)** Representative images of CD31-positive structures and calculation relative to the total wound area outlined in yellow. **(C–E)** Frequencies of **(C)** CD31+, **(D)** CD68+ and **(E)** CD163+ cells were determined by immunohistochemical staining relative to the total wound area. **(F–H)** Histological wound healing indices: **(F)** Epithelial thickness index (ETI) calculated by comparing epithelial thickness in the uninjured skin and wounded area, **(G)** scar elevation index (SEI) calculated by comparing the dermis thickness in the uninjured skin and wounded area and **(H)** collagen density after Azan staining in the uninjured skin and wounded area. Quantification was done using QuPath algorithms. Data from individual wounds are shown. *p≤ 0.05, **p≤ 0.01, ***p ≤ 0.001, **** p ≤ 0.0001, as calculated using one-way ANOVA und Tukey-Test.

Having observed accelerated wound healing in hMSCs-treated wounds, histological analysis was performed. To gain insight into cell infiltration to the wounds, cells in total, lymphocytes and fibroblasts were identified based on their typical nuclear and cell phenotypic features (**Figure 6A**). In the hMSCs-treated wounds more lymphocytes could be detected compared to untreated and fibrin glue-treated wounds, whereas fibroblast and total cell numbers seemed unaffected by hMSCs treatment (**Figure 6A**).

**Figure 7:**
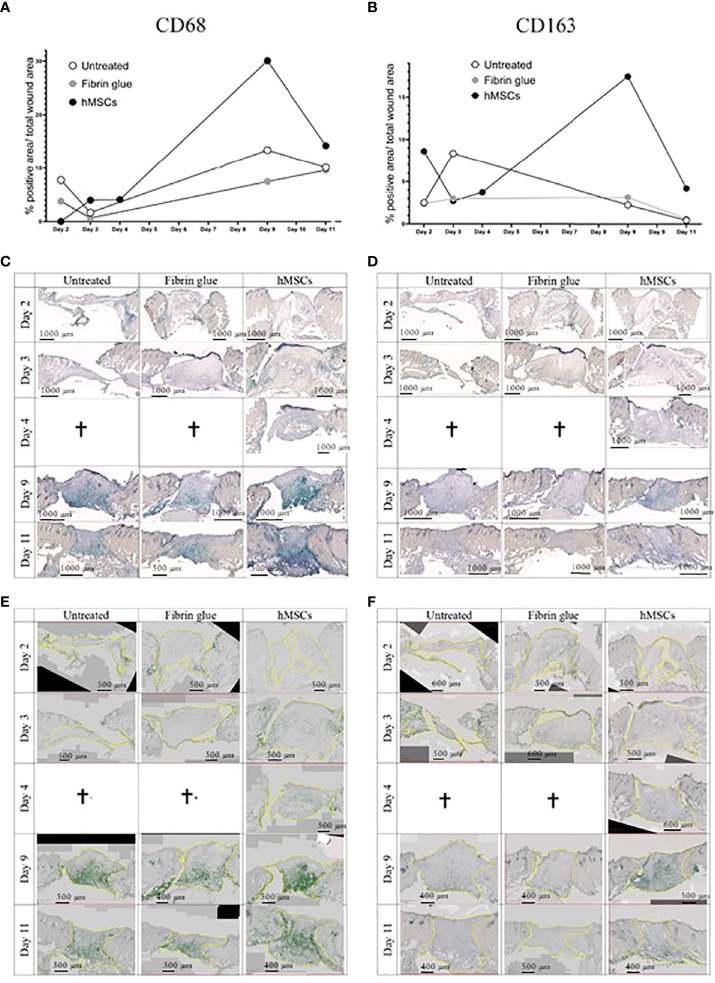
hMSCs rapidly recruit macrophages, infiltrating wounds from the wound edges and basis. Immunohistochemical staining of **(A, C, E)** CD68- and **(B, D, F)** CD163-positive macrophages. **(A, B)** Quantification as described in Figure 4. **(C, D)** Representative images are shown. **(E, F)** Wound margins are indicated in yellow, the histogreen-positive signal is highlighted and the histogreen-negative background signal reduced to visualize macrophage recruitment kinetics and routes.

In a next step, immunohistochemical staining of CD31, indicative of tissue vascularization, CD68 as pan-macrophage marker and CD163 as marker for M2-subtype anti-inflammatory macrophages, activated in murine wound healing promoting to anti-inflammatory functions, extracellular matrix formation and angiogenesis (55), was performed. We found an increase in CD31+ capillaries in the wounds repeatedly treated by hMSCs doses compared to untreated controls and fibrin glue-treated controls (**Figures 6B, C**). The hMSCs-treated wounds showed also a higher proportion of CD68+ and CD163+ infiltrated macrophages, compared to both control groups (**Figures 6D, E**). Detailed microscopic wound assessment at different time points after wound setting (part of the biodistribution study) revealed gradual infiltration of macrophages from the wound edges (d3), then the basal wound area (d9), eventually progressing to the apical wound tissue on d11 (**Figure 7**). In the hMSCs-treated wounds, CD68+ cell infiltration extended more towards the apical layers than in both controls (**Figures 7C, E**). This infiltration was accelerated in hMSCs-treated wounds at day 9 to then drop to similar levels as both controls on d11 (**Figure 7A**). Starting at day 4, hMSCs promoted infiltration/differentiation of CD163-expressing macrophages, whereas both control-treated wounds revealed no increase in CD163-positive cells. Only in hMSC-treated wounds the CD163+ signal peaked at day 9 (**Figure 7B**) with positive signals in the entire wound area extending to the apical layer (**Figures 7D, F**).

### hMSCs improve epithelial thickness, reduce scar elevation and increase collagen density in healing wounds

Having documented that hMSCs led to an increase in vascularization and induced CD68-, but also CD163-positive macrophage infiltration, we aimed to gain more insights into the healing dynamics of the wounds. Here, a histology scoring system was used (39). First, an epithelial thickness index was calculated (**Figure 6F**). Almost all wounds demonstrated hypertrophy of the epithelium, indicative for their healing stage (39). Without statistical significance, mean values suggested that the hMSCs-treated wounds showed the least epithelial thickness, followed by fibrin and then untreated wounds (**Figure 6F**). Second, a scar elevation index was calculated. All wounds demonstrated hypoplasia of the dermis, yet hMSCs-treated wounds were already close to a normal state, significantly different to the untreated wounds, indicating an already better-developed wound compared to both controls (**Figure 6G** **).** Third, collagen density was calculated based on intensity of blue Azan stain and compared to the respective non-wounded dermis. After migration into the wound, fibroblasts gradually produce ECM and collagen fibers. During wound healing, especially during proliferation stage, collagen accumulates in wounds, resulting in a darker blue Azan stain. Our results indicated a significantly higher collagen deposition and density in hMSCs-treated wounds on day 14 of our experiment compared to controls indicative of improved collagen deposition (**Figure 6H**).

### hMSCs are only transiently detectable in wounds

To assess the fate and biodistribution of topically applied hMSCs over time within the wounds and in distant organs, animals were sacrificed on day 1, 2, 3, 4, 9 and day 11, followed by histological as well as dPCR-based quantification of human cells.

**Figure 8:**
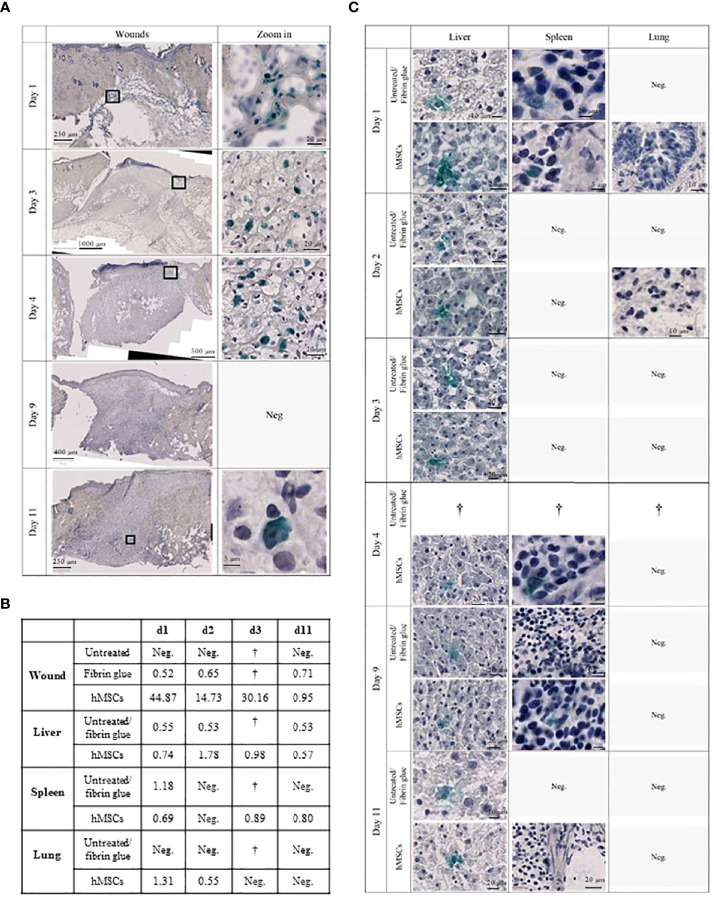
hMSCs are only transiently detectable in wounds. **(A)** Human Ku80-Histogreen staining in wound cross-sections. Left: Cross section of the entire wound. Right: Zoom on hKu80-positive cells. On day 1, wound edges with fibrin glue containing hMSCs located under the intact dermis are shown. Day 3 and 4 after hMSCs application, intact hMSCs were located in the fibrin glue top of the wound. Despite detection of human DNA in the wounds by dPCR on day 9, no hKu80 signal was detected in the histological sections. On day 11, very few hKu80-positive cells were found in the basal part of the wounds. **(B)** dPCR results of human DNA in rat wounds and organs. A value of ≥ 0.5 copies/µl was taken as positive result. **(C)** hKu80 expression in cryosections of analyzed organs: representative microphotographs are shown for samples where human cells were detected; no microphotographs are shown for negative samples.

Staining the wound sections for human nuclear Ku80 expression (38), we identified topically applied hMSCs on day 3 and 4 within the area of the fibrin glue interspersed within non-human tissue, yet hMSCs were undetectable at later time points **(** **Figure 8A** **)**.

Accordingly, we confirmed the presence of human DNA in hMSCs-treated wounds. Levels decreased over time suggesting that the hMSCs were gradually eliminated from the wounds (**Figure 8B**). Interestingly, traces of human DNA were also detected in cell-free fibrin glue-treated wounds, but never in untreated wounds, suggesting that the fibrin glue might contain low levels of human DNA. Human DNA was also detected in the livers of hMSCs-treated rats on day 1, 2, 4 and 11. The histological crosscheck revealed that the hKu80 signal was located in the cytoplasm, but not in the nucleus of the rat hepatocytes (**Figure 8C**).

We also detected traces of human DNA in livers of rats having one of their wounds treated with fibrin (**Figure 8B**). These results were confirmed by histological analysis. Again, the hKu80 stain was cytoplasmic (**Figure 8C**). It appears that not only hMSCs, but also the fibrin glue fragments, to a lesser extent, were transported from the wound site to the liver for phagocytosis by hepatocytes. Traces of human DNA were also found in the spleen of hMSCs-treated rats on days 1, 4 and 11 and in the spleens of animals with a fibrin glue-treated wound on day 1. Histological analysis confirmed this result as well (**Figure 8C**). On day 1 and 2, dPCR detected the presence of human DNA in the lungs of hMSCs-treated rats. No human DNA was found in the lungs of fibrin glue-treated rats.

## Discussion

A well-standardized MSC therapeutic can improve the validity of clinical studies. Yet, the transfer of lab-scale protocols for hMSC manufacture to sustainable clinical adoption is laden with technical obstacles, issues pertaining to hMSC biology and variable MoAs (13). Here, we propose a GMP-compliant protocol, which allows for scalable and reproducible manufacturing of qualified clinical hMSCs batches at minimal *in vitro* expansion. All steps performed use only one batch of pooled pathogen-inactivated platelet lysate (25). We demonstrate that these pooled hMSCs, when topically applied, are potent in accelerating wound healing in a preclinical rat model without systemic effects. In this decision-making approach, we prepare the grounds for clinical wound healing studies: i) apply hMSC topically in a fibrin glue matrix, ii) preferably use fresh hMSCs, and iii) perform repeated application.

**Figure 9:**
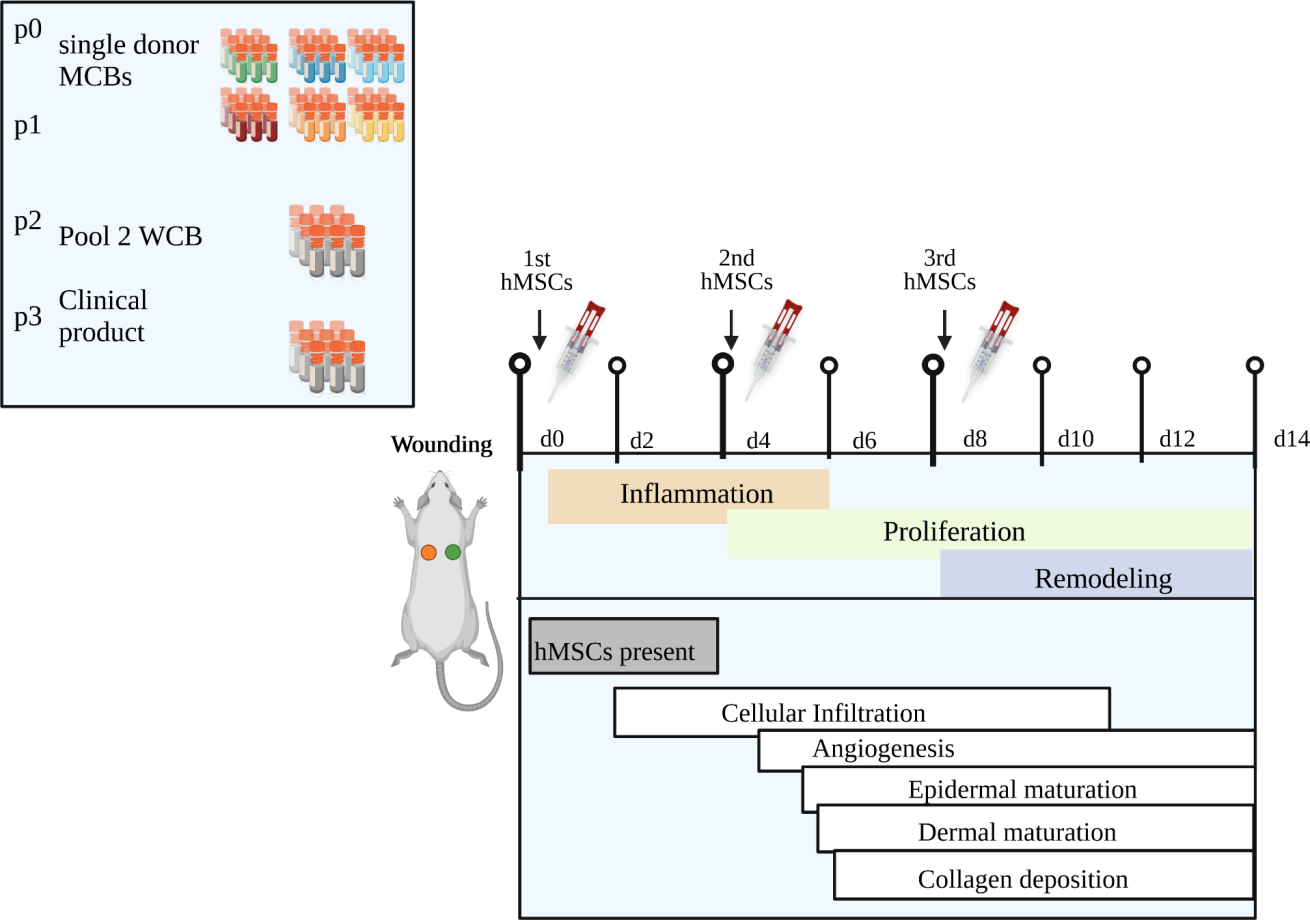
Summary of key findings. Our novel clinical-scale manufacturing concept is comprised of six single donor hMSCs master cell banks that are pooled to a working cell bank from which an extrapolated number of 70,000 clinical doses of 1x10^6^ hMSCs/cm^2^ wound size can be manufactured within only three passages. Repeated topical hMSCs administration significantly accelerated the wound healing in a diabetic rat model by delivering a defined growth factor cargo at the specific stages of wound repair, namely inflammation, proliferation and remodeling. Specifically, the hMSCs mediated epidermal and dermal maturation and collagen formation, improved vascularization, and promoted cell infiltration, especially a dynamic recruitment of M2 macrophages. Created with BioRender.com.

Donor-to-donor variability and extensive *in vitro* expansion are major drivers of inconsistent results from clinical trials, particularly when hMSCs batches are manufactured from single donors (3, 56). Pooling single donations, a concept implemented for platelet concentrates for many years (57), has been introduced recently for both hMSC and hPL products (13, 16, 19, 20, 25, 58). To balance the needs for scale-up and low-level expansion of a pooled hMSC product, our concept starts with MCBs from single donor hMSCs. From these, pooled hMSCs WCBs can be repeatedly manufactured, thus maximizing the starting material for clinical doses within only three passages (**Figure 9**). We argued about the best pooling time point. Contrary to “MSC-FFM”, pooled as MNCs, and “Stempeucel^®^”, pooled at passage 1 followed by five to six expansion passages (17, 20), we compared pooling at passage 1, 2 and 3. As expected, the *in vitro* tests showed that quality did not differ significantly between the different hMSC pools. Pool 2 was chosen based on the potential to produce up to 70,000 clinical doses (1x10^6^ hMSCs/cm^2^ wound size) at minimal *in vitro* expansion burden.

Exposure to multiple donors, however, may increase the risk of transmitting infectious agents. We propose to address this by i) rigorous donor testing according to blood banking standards (e.g. individual donor nucleic acid testing for HIV, HBV, HCV with increased sensitivity), by ii) pooling only a limited number of donors (we decided on six), and iii) a thorough MCB evaluation that includes re-testing for infectious agents. Our concept of six separate single donor MCBs and one pooled hMSCs WCB ascertains individual release testing of each cell bank. This ensures that the hMSCs from each donor are re-tested before being formulated as clinical product. For unknown infectious agents, though, a risk remains. In general, this pertains to pooled hPL as well. Here, however, PRT can be applied (22). In this case, we used hPL treated by high-dose gamma irradiation. The large hPL batch we used in this study was pooled from 70 single donors, allowing to produce this substantial number of clinical hMSC doses and for leveling-out single hPL donor variances (25).

Furthermore, we scaled-up the manufacturing process by expanding the pooled hMSCs in a closed cell culture system, where all media change and harvest steps are performed *via* sterile-connected bags. This reduces the hands-on time as well as open handling steps and allows direct transfer to clinical production according to GMP.

Successfully having addressed challenges of hMSC product manufacturing, we next moved to testing their preclinical efficacy in a diabetic wound healing rat model, known for their impaired wound healing capacity reflecting chronic healing defects in patients. The wound healing process is composed of three overlapping phases: inflammation, proliferation and remodeling (5). Pro-inflammatory macrophages, simplified referred to as M1 macrophages, infiltrate the wound at first to sanitize it from debris. In healing wounds, anti-inflammatory and pro-regenerative macrophages (M2) take over mediating migration and proliferation of fibroblasts, keratinocytes, and endothelial cells to restore dermis, epidermis and vasculature (55). Chronic wounds fail to heal because they remain in the early inflammation phase. We show that pooled hMSCs accelerated wound healing with significant reduced wound sizes already at day 4. Assuming that hMSCs mediate wound repair by delivering their trophic factors cargo, we calculated the trophic factors content per dose of the hMSC product, eventually allowing for a correlation between the trophic factor content in the clinical product and the strength of its therapeutic efficacy. Our hMSC product contains a variety of trophic factors known to promote the different phases of wound healing at stable concentrations in different batches. Repeated treatment with pooled hMSCs promoted cell infiltration and M2 macrophage recruitment, improved vascularization and induced epidermal and dermal maturation and collagen formation. We propose that the repeated hMSCs application not only increases the cumulative factors dose, but also importantly provides the different factors each at the right time during the different wound healing phases. G-CSF, IL-1α, IL-6, TGF-β, PGE-2 and inducible IDO-1 likely contributed to the observed immune infiltration and the consequent important inflammation phase. As per their known functions, TGF-β, PGE-2 and LIF mediate dynamic recruitment and polarization of macrophages. Further, it is reasonable to assume that VEGF-1, FGF-2, BDNF and HGF are responsible for the increased vessel density, and EGF, HGF, IL-1α, osteopontin and FGF-2 for keratinocyte and fibroblast proliferation/differentiation and ECM remodeling as indicated by the improved epithelial thickness, scar elevation and collagen density (**Figure 9**).

To the best of our knowledge, we describe for the first time in detail the dynamics of macrophage recruitment and polarization in hMSC-treated wounds. Specifically, hMSCs recruited more CD68+ and CD163+ macrophages gradually into all wound layers, first from the wound edges (d3), then the basal wound area (d9), finally progressing to the apical wound tissue on d11. This indicates an increased motility of these recruited macrophages. By chemotactically attracting macrophages and polarizing them to pro-regenerative M2 macrophages, hMSCs orchestrate the dysbalanced immune response within the wound (55, 59, 60).

We detected hMSCs in the wounds for up to 4 days by both dPCR and hKu80 nuclear staining. Yet, they apparently failed to persist and to differentiate *in situ* into endothelial cells or keratinocytes, as observed previously (8, 61). However, we found traces of human DNA in various organs already one day after topical application, of note, both in hMSCs-treated and human fibrin glue-treated animals. Histological staining verified the presence of hKu80 protein in these organs, confirming the dPCR results. This may indicate that human DNA and protein were removed from the wounds by phagocytes and then rapidly distributed to lung, spleen and liver. Yet, we cannot exclude that few intact human cells may have found their way to these organs. Here, however, the Ku80 signal was located in the cytoplasm. This may suggest that these human cells were oxidatively stressed (62).

The transient presence of hMSCs within the wounds could be explained by macrophage-mediated immunological clearance of xenogeneic hMSCs. In fact, Galleu et al. suggested the efferocytosis of allogeneic hMSCs as key for clinical efficacy, at least in the context of GvHD (63). In the GvHD setting, only those patients capable of immunologically clearing hMSCs benefitted from the hMSC therapy.

An adverse immune response may occur after repeated application of allogeneic or xenogeneic cells. A recent trial evaluating intravenously injected ABCB5+ allogeneic skin-derived hMSCs in patients with epidermolysis bullosa reported two patients with severe, yet transient and manageable, hypersensitivity reactions (11). We, however, even after repeated topical application (three subsequent doses), found no or only mild signs of an adverse immune response in our preclinical model, similar to previous studies (10, 64, 65). Ardanaz et al. conclude that once no hypersensitivity response to a second pooled allogeneic BM-MSC injection is observed, repeated treatments are possible to potentiate the benefit of hMSC therapy (66).

Given that hMSCs exert their therapeutic MoAs on various levels, we propose a matrix of potency assays for product release, combining trophic factors concentrations (min/max ranges) with functional and quality control tests that predict clinical efficacy. An example for this matrix approach is the measure of i) IL-1RA secretion in response to stimulation by M1-polarized macrophages, ii) pro-angiogenic VEGF secretion after 48h hypoxia, and iii) tube formation of hMSCs on matrigel as potency test matrix for ABCB5+ skin-derived hMSCs for treatment of chronic venous ulcers, epidermolysis bullosa, and liver disease (67).

For purity testing, we used binary marker expression as typically assessed by flow cytometry. Our hMSCs, single donor- and pool-derived, followed the consented hMSCs binary marker profile (33). To predict immunomodulatory capacity, we documented their T cell inhibitory potential and inducible IDO-1 expression with highly reproducible results. As novel assays, we introduced an angiogenesis support assay and further, to control hMSCs fitness, a scratch wound healing and a chemotaxis assay. Yet, for these three assays day-to-day and operator-to-operator-variations were apparent calling for better standardization. A cell ruler could improve individual assay run reproducibility and thus improve batch-release testing (68). The need for thorough in-house protocol standardization, but also transfer and training to other sites, is confirmed by a recent multicenter study, which reports that the factor “production site” contributed more to variations in hMSC cultures than the source material used for hMSC production (69).

Within this decision making study, we prepared the fundament for a clinical study assessing pooled hMSCs efficacy in chronic wound healing. We not only provide data of a scalable manufacturing protocol, but also evidence for preclinical efficacy of the pooled hMSC product acting on different phases of wound healing. We reproduced a protocol for clinical administration of hMSCs to the wounds using fibrin glue. In the preclinical model with relatively small wounds, we applied the hMSCs topically in fibrin glue *via* a syringe. However, Falanga et al. already showed that for large wounds this administration works even well when applied as spray (8). Besides providing a stable matrix for the hMSCs, fibrin clot formation is an essential component of physiological wound healing. We speculated that the cell-free fibrin glue might per se improve wound healing. Yet, this was not observed in the ZDF model, possibly related to the 1:10 dilution. Interestingly, fibrin glue-treated wounds showed increased crust formation compared to hMSCs-fibrin glue-treated wounds. As ZDF rats are known for impaired wound contraction, increased inflammation and abundant crust production (31), the reduced crust formation in the hMSC group may be attributed to their known fibrinolytic activity (70). Furthermore, improved crust degradation may indicate the accelerated wound healing by hMSCs.

Given that preclinical models lack to reflect fully the complexity of human chronic non-healing wounds, well-designed clinical trials are required. We suggest a manufacturing protocol yielding in hMSCs of proven biological potency that can be instantly manufactured as an off-the-shelf product at clinical scale. As a pooled product, it levels-out donor heterogeneity. It further allows for scaled and reliable production of standardized clinical cell batches, based on the MCB/WCB concept and the use of a pathogen-inactivated pooled hPL. We confirm data that hMSCs applied in a fibrin glue are therapeutically active in accelerating wound healing, best when obtained from a rescue-culture and applied repeatedly.

In conclusion, we provide scientific evidence for a standardized, scalable and, importantly, efficacious pooled hMSC product. We show that these pooled hMSCs with a defined wound healing factor cargo accelerated the dermal wound healing in diabetic rats by improving vascularization and dynamically recruiting M2-like macrophages (**Figure 9**). The next steps to acquire a manufacturing license as an advanced therapy medicinal product (ATMP) for clinical use, are i) validate the *in vitro* assays for batch qualification and release testing, ii) perform a thorough preclinical biodistribution, toxicity, and tumorigenicity study program and iii) finally a clinical trial.

## Data availability statement

The raw data supporting the conclusions of this article will be made available by the authors, without undue reservation.

## Ethics statement

The studies involving human participants were reviewed and approved by 329/10, ethics committee, University Hospital Frankfurt am Main, Germany. The patients/participants provided their written informed consent to participate in this study. The animal study was reviewed and approved by G142-19, Regierungspräsidium Karlsruhe, Germany.

## Author contributions

All authors contributed to gathering of data, writing, editing, and revising of the manuscript.

## Funding

We acknowledge the financial support of the German Red Cross Blood Donor Service Baden-Württemberg - Hessen. This work was supported by a grant from the Deutsche Forschungsgemeinschaft (DFG GRK 1874-2 DIAMICOM, SP6 – HW and KB). For the publication fee we acknowledge financial support by Deutsche Forschungsgemeinschaft within the funding programme “Open Access Publikationskosten” as well as by Heidelberg University.

## Acknowledgments

We acknowledge the support from Stefanie Uhlig, Flow Core Mannheim, Core Facility Platform Mannheim (CFPM), Silke Vorwald, Institute of Neuroanatomy, Dr. Bettina Kränzlin and the entire core facility Preclinical Models and the LIMa Live Cell Imaging Mannheim core, both CFPM, Medical Faculty Mannheim, Heidelberg University.

## Conflict of interest

BD and MG are employees of Macopharma.

The remaining authors declare that the research was conducted in the absence of any commercial or financial relationships that could be construed as a potential conflict of interest.

 The authors declare that this study received funding from Macopharma. The funder had the following involvement in the study: provision of funding and material; manuscript writing and review of the final manuscript. Macopharma also provided high-dose gamma irradiated pooled hPL.

## Publisher’s note

All claims expressed in this article are solely those of the authors and do not necessarily represent those of their affiliated organizations, or those of the publisher, the editors and the reviewers. Any product that may be evaluated in this article, or claim that may be made by its manufacturer, is not guaranteed or endorsed by the publisher.

## Supplementary material

Supplementary Figure 1 All hMSC pools display similar adipogenic and osteogenic differentiation potential. hMSCs were seeded at a density of 1,000 cells/cm^2^ and grown to subconfluency. Adipogenic differentiation was induced using the hMSC Adipogenic Differentiation Medium BulletKit (Lonza), or osteogenic differentiation was induced using osteogenic medium composed of α-MEM/10% FBS supplemented with 1 μM dexamethasone, 50 μM ascorbic acid, and 10 mM β-glycerolphosphate (Sigma–Aldrich). After 3 weeks under differentiation conditions, lipid vacuoles in adipogenic cultures were stained with oil red O and calcium deposits of osteogenic cultures with alizarin red S, respectively.

Supplementary Figure 2 hMSCs migrate out of diluted fibrin glue. Instead of being applied to a wound, hMSCs were seeded in fibrin glue into a cell culture well plate and cultivated in medium supplemented with hPL versus serum-free medium as control. 4.5 hours post-seeding, hMSCs migrated from the glue attaining their typical fibroblast-like morphology. hMSCs in serum-free medium showed no migration out of the gel. (Axio Vert.A1, 5-fold magnification).

Supplementary Figure 3 No systemic wound healing effect after topical hMSCs application. Rats were wounded and hMSCs applied topically in diluted fibrin glue. Comparison of wound size reduction of contralateral untreated wounds with lateral either **(A)** untreated, **(B)** hMSCs cryo single application, **(C)** fibrin glue single application, **(D)** single application hMSCs fresh, **(E)** repeated application of fibrin glue or **(F)** repeated application of hMSCs fresh d0, 4 and 8. Quantification of the wound area was performed with Image J. ** p≤ 0.01, as calculated using two-way ANOVA und Sidak-Test.

Supplementary Table 1 Trophic factors content in hPL MultiPL’100i (batch 11219267DM), including batch release test results. Experiments performed on two different bags (#209 and 221) from the used MultiPL’100i batch 11219267DM, means and standard deviations are shown. ^1^ Experiments repeated twice with different controls and verified using ELISA kits from two different companies. bFGF, basic fibroblast growth factor; IGF, insulin-like growth factor 1; TGF-β1, transforming growth factor beta 1; EGF, epidermal growth factor; PDGF-AB, platelet-derived growth factor AB; VEGF, vascular endothelial growth factor; TNF-*a*, tumor necrosis factor alpha; IFN-γ, interferon gamma; LAL, limulus amebocyte lysate; NTU, nephelometric turbidity unit.
