# Supplementary figures and images for: Pooled human bone marrow-derived mesenchymal stromal cells with defined trophic factors cargo promote dermal wound healing in diabetic rats by improved vascularization and dynamic recruitment of M2-like macrophages
Source: Front Immunol. 2022 Aug 19;13:976511. doi: 10.3389/fimmu.2022.976511 (PMC9437960; doi:10.3389/fimmu.2022.976511)

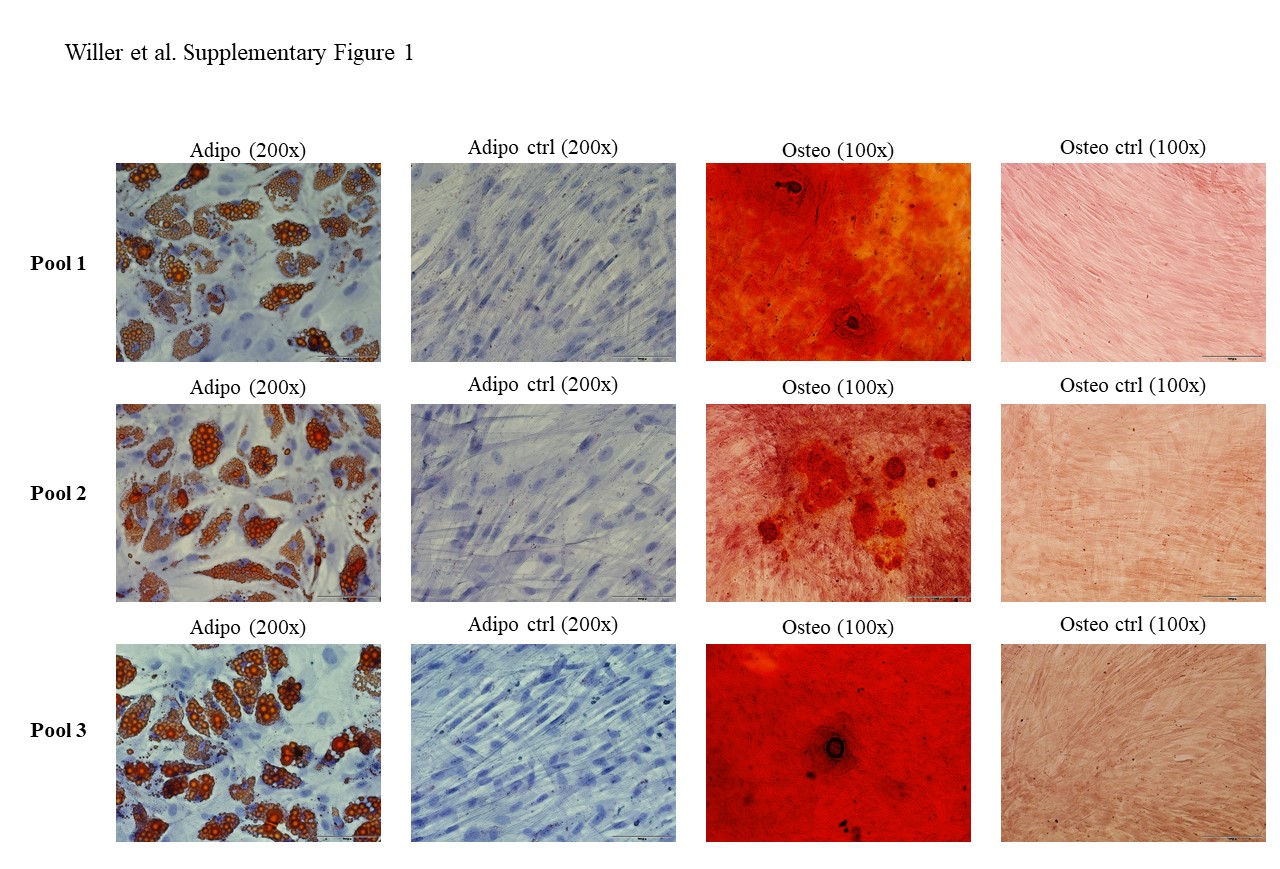

Supplement: Supplementary Figure 1 — All hMSC pools display similar adipogenic and osteogenic differentiation potential. hMSCs were seeded at a density of 1,000 cells/cm2 and grown to subconfluency. Adipogenic differentiation was induced using the hMSC Adipogenic Differentiation Medium BulletKit (Lonza), or osteogenic differentiation was induced using osteogenic medium composed of α-MEM/10% FBS supplemented with 1 μM dexamethasone, 50 μM ascorbic acid, and 10 mM β-glycerolphosphate (Sigma–Aldrich). After 3 weeks under differentiation conditions, lipid vacuoles in adipogenic cultures were stained with oil red O and calcium deposits of osteogenic cultures with alizarin red S, respectively. [file Image_1.jpeg]

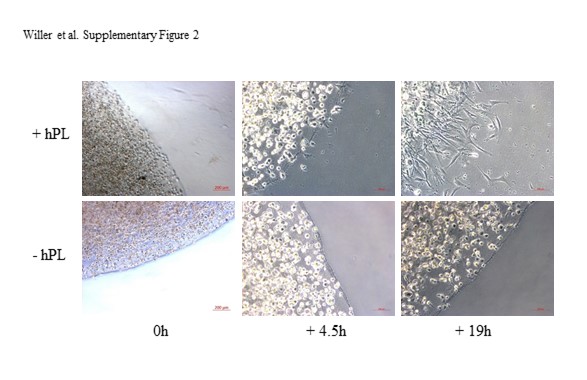

Supplement: Supplementary Figure 2 — hMSCs migrate out of diluted fibrin glue. Instead of being applied to a wound, hMSCs were seeded in fibrin glue into a cell culture well plate and cultivated in medium supplemented with hPL versus serum-free medium as control. 4.5 hours post-seeding, hMSCs migrated from the glue attaining their typical fibroblast-like morphology. hMSCs in serum-free medium showed no migration out of the gel. (Axio Vert.A1, 5-fold magnification). [file Image_2.jpeg]

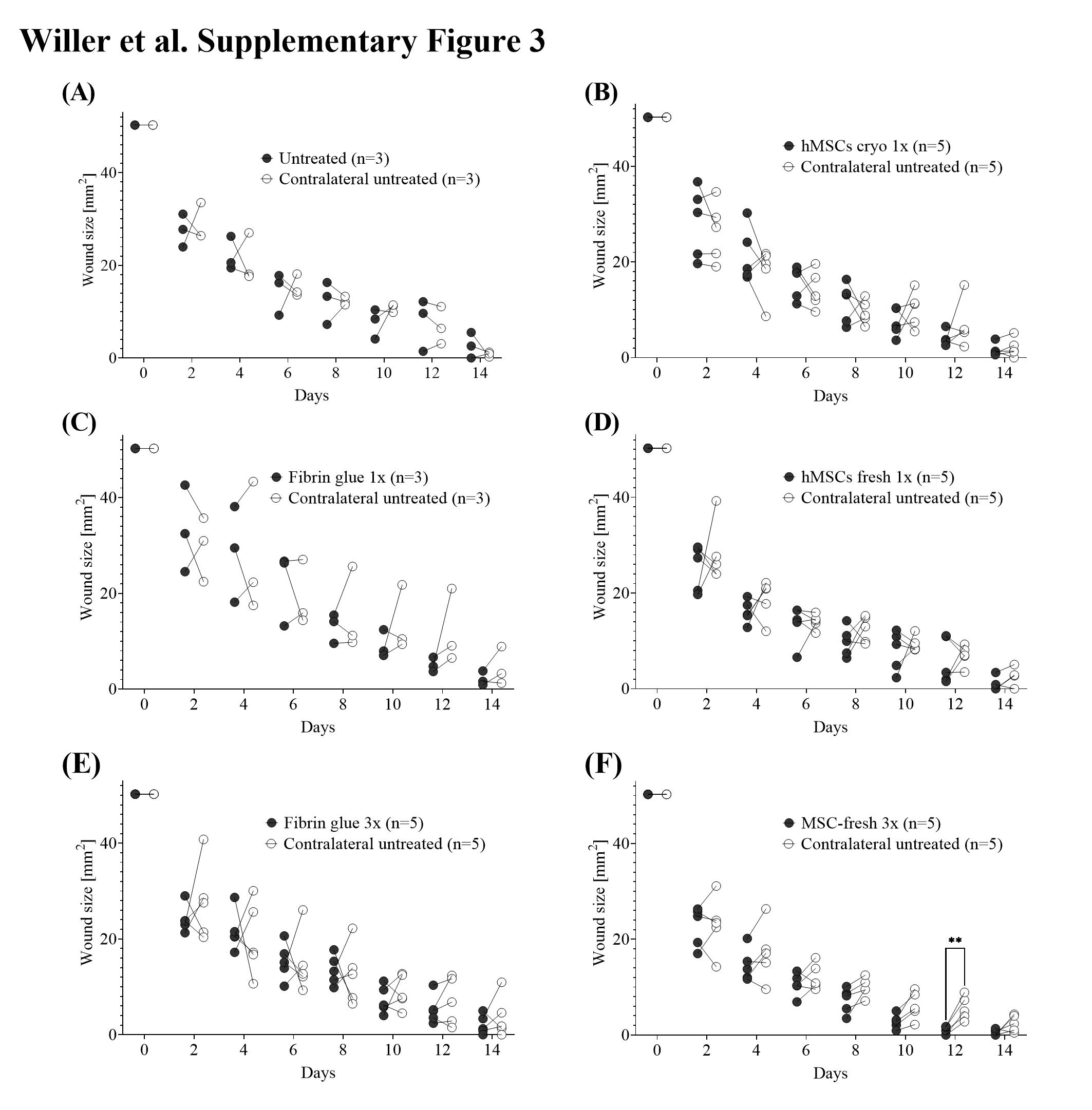

Supplement: Supplementary Figure 3 — No systemic wound healing effect after topical hMSCs application. Rats were wounded and hMSCs applied topically in diluted fibrin glue. Comparison of wound size reduction of contralateral untreated wounds with lateral either (A) untreated, (B) hMSCs cryo single application, (C) fibrin glue single application, (D) single application hMSCs fresh, (E) repeated application of fibrin glue or (F) repeated application of hMSCs fresh d0, 4 and 8. Quantification of the wound area was performed with Image J. ** p≤ 0.01, as calculated using two-way ANOVA und Sidak-Test. [file Image_3.jpeg]
