# Supplementary material for: Pooled human bone marrow-derived mesenchymal stromal cells with defined trophic factors cargo promote dermal wound healing in diabetic rats by improved vascularization and dynamic recruitment of M2-like macrophages
Source: Front Immunol. 2022 Aug 19;13:976511. doi: 10.3389/fimmu.2022.976511 (PMC9437960; doi:10.3389/fimmu.2022.976511)
Supplement: Supplementary Table 1 — Trophic factors content in hPL MultiPL’100i (batch 11219267DM), including batch release test results. Experiments performed on two different bags (#209 and 221) from the used MultiPL’100i batch 11219267DM, means and standard deviations are shown. 1 Experiments repeated twice with different controls and verified using ELISA kits from two different companies. bFGF, basic fibroblast growth factor; IGF, insulin-like growth factor 1; TGF-β1, transforming growth factor beta 1; EGF, epidermal growth factor; PDGF-AB, platelet-derived growth factor AB; VEGF, vascular endothelial growth factor; TNF-a, tumor necrosis factor alpha; IFN-γ, interferon gamma; LAL, limulus amebocyte lysate; NTU, nephelometric turbidity unit. [file Table_1.pdf]

| hPL MultiPL'100i | |
|---|---|
| **ELISA** | **pg/ml** |
| bFGF | 85.43 ± 1.88 |
| IGF-1 | 95.34 ± 4.63 |
| TGF-*b*1 | 53647.50 ± 194.45 |
| EGF | 2287.7 ± 43.84 |
| PDGF-AB | 34257.00 ± 1032.38 |
| VEGF | 608.41 ±13.30 |
| TNF-α [1] | below detection limit |
| IFN-ɣ [1] | below detection limit |
| **batch release tests** | |
| Endotoxins (LAL) | conform<1UI/mL |
| Osmolality | 292 mOsmol/kg H2O |
| Turbidity | 140 NTU |
| pH | 7,5 |
